# Pool-Type Fishways: Two Different Morpho-Ecological Cyprinid Species Facing Plunging and Streaming Flows

**DOI:** 10.1371/journal.pone.0065089

**Published:** 2013-05-31

**Authors:** Paulo Branco, José M. Santos, Christos Katopodis, António Pinheiro, Maria T. Ferreira

**Affiliations:** 1 CEF – Centro de Estudos Florestais, Instituto Superior de Agronomia, Universidade Técnica de Lisboa, Lisboa, Portugal; 2 Katopodis Ecohydraulics Ltd., Winnipeg, Manitoba, Canada; 3 CEHIDRO – Centro de Estudos de Hidrossistemas, Instituto Superior Técnico, Universidade Técnica de Lisboa, Lisboa, Portugal; Pacific Northwest National Laboratory, United States of America

## Abstract

Fish are particularly sensitive to connectivity loss as their ability to reach spawning grounds is seriously affected. The most common way to circumvent a barrier to longitudinal connectivity, and to mitigate its impacts, is to implement a fish passage device. However, these structures are often non-effective for species with different morphological and ecological characteristics so there is a need to determine optimum dimensioning values and hydraulic parameters. The aim of this work is to study the behaviour and performance of two species with different ecological characteristics (Iberian barbel *Luciobarbus bocagei–*bottom oriented, and Iberian chub *Squalius pyrenaicus–*water column) in a full-scale experimental pool-type fishway that offers two different flow regimes*–*plunging and streaming. Results showed that both species passed through the surface notch more readily during streaming flow than during plunging flow. The surface oriented species used the surface notch more readily in streaming flow, and both species were more successful in moving upstream in streaming flow than in plunging flow. Streaming flow enhances upstream movement of both species, and seems the most suitable for fishways in river systems where a wide range of fish morpho-ecological traits are found.

## Introduction

The increasing demand of water for human consumption propels the construction of dams and weirs that become insurmountable barriers to the migration of fish, thus imperiling the completion of the life cycle of several fish species. Indeed, more than half of the world’s largest rivers are currently negatively impacted by dams and weirs [1], which have caused serious declines of both resident and migratory fish populations [2] by affecting upstream adult migration, reproduction, feeding and colonization movements, while promoting genetic impoverishment and dispersion of exotic species [3].

To overcome this problem, barriers must become negotiable by fish. To achieve this goal, the construction of fish transfer devices, commonly known as fishways, has been considered the most feasible measure to improve and restore connectivity. The problem with this measure is the often low efficiency of such devices for weak swimming species [4]. Fishways have traditionally been constructed based on guidelines developed and tested for salmonids, known strong swimmers with great leaping abilities, while studies of fishway performance focused on species with low economic and recreational value (e.g. cyprinids) continue to be neglected [5]. This is rather unfortunate, since these species are an important biological component of fish assemblages and free instream movement is indispensable for their survival [6]. In addition, recently, several non-salmonid species have acquired greater legislative protection (e.g. under the EU Habitats Directive [7]).

It is a complex problem to assure that flow and turbulence conditions within a fishway provide flow patterns suitable for an array of species. Pool-type fishways are a very common type of fish transfer device that has been built since the nineteenth century [8]. They consist of a rectangular flume divided by cross-walls that create a series of consecutive pools arranged in a stepped pattern each upstream from the preceding one. The purpose of these pools is to divide the height to be negotiated by fish, while ensuring no kinetic energy of the jets coming from the bottom orifices or the notches is transmitted to the downstream basins. This creates similar flow patterns in each pool [8]. Additionally, these pools offer resting areas for fish to recover after negotiating the cross-walls. The cross-walls between the pools may be equipped with different opening types - surface notches and submerged orifices at the bottom – that are used by the fish to move from pool to pool. The selection of an opening type by a fish depends on the species swimming ability and on the flow regime passing through the device [9] plunging or streaming [10]. In the plunging flow regime, the water level in the pool immediately below the cross-wall (producing the plunging flow) is below the crest of the notch; in the streaming flow mode, a surface stream appears to flow over the crest of the notches, skimming over the water surface of the pools in between (further details in: [11], [12]). Pool-type fishways, which include pool and weir and vertical slot, are the most efficient conventional or technical fishway types constructed, either for salmonids or for non-salmonids [4]. Only fishways with optimal design can be of high efficiency as their success varies according to swimming ability, size [13]–[15] and physiologic state [16], [17] of different fish species, as well as hydraulics [18] and turbulence [19]. The design criteria for pool-type fishways are quite well understood for diadromous species [9], [11], [18]. There is still a knowledge gap though on how to improve and make these passes more efficient and more capable to accommodate a wider range of species and size-classes. This is particularly important for potamodromous and resident cyprinid species, as international environmental legislation requirements are increasingly more stringent for species [18], [20].

Fish species evolved differently, to be adapted to different riverine environments. These specializations can be grouped into different morpho-ecological guilds that introduce differences in swimming abilities, behaviour and niche occupancy. The best method to understand the influence of these differences on fishway negotiation ability is to test different species, representative of different morpho-ecological guilds, on fishways with controlled conditions. Laboratory trials, where conditions found in the field are easily reproduced while manipulating variables and monitoring confounding factors are always preferable [21], and have been proposed as the starting point of successful fishway designs [18], [22].

The purpose of this work is to study the behaviour and performance of cyprinid species with different ecological characteristics, in a full-scale indoor model of a pool-type fishway, fitted with bottom orifices and surface notches while subjected to one of two flow regimes - plunging and streaming. Hence, two species were used, *Luciobarbus bocagei* (Steindachner, 1864) a large-bodied potamodromous benthic fish, and *Squalius pyrenaicus* (Günther, 1868) a small-bodied water-column resident fish. It is thus hypothesized that: (1) the proportion of upstream movements through surface notches and submerged orifices will vary depending on the species and flow regime type; (2) both species will use the submerged orifices in greater proportions during the plunging flow regime, and (3) fish upstream movements and successes (when a fish reaches the top of the fishway by negotiating the fifth cross-wall) will be higher for both species during streaming flow conditions.

## Materials and Methods

### Ethics Statement

Animal trials and sampling were conducted in agreement with national and international guidelines to maintain wellfare of the individuals [23], [24]. Fish sampling permits were obtained from the National Forest Authority. The experiments were carried out in strict accordance with the recommendations of the “protection of animal use for experimental and scientific work” of the Department for Health and Animal Protection (Direcção de Serviços de Saúde e Protecção Animal) that authorized animal experiments to be conducted in this experimental facility, and fish to be maintained in the laboratory (permit number: 16546–7/10/2011). All efforts were made to minimize stress and no fish were sacrificed to complete this study.

### Fishway Facility

**Figure 1:**
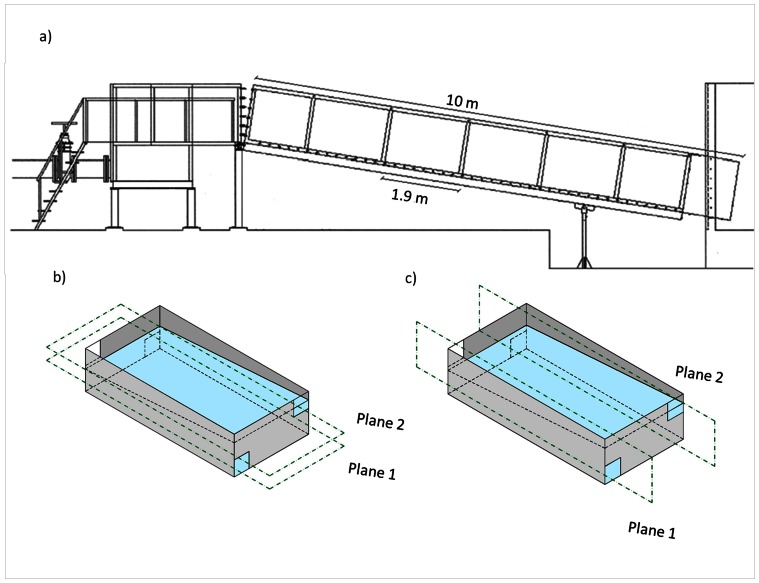
Schematic view of the experimental prototype and measurement planes. a) Side view diagram of the pool-type fishway experimental prototype on a slope of 8.5%; b) Three dimensional representation of a pool, showing orifice arrangements and the horizontal planes (dashed green lines) where hydraulic measurements were conducted; c) Three dimensional representation of a pool, showing orifice arrangements and the vertical planes (dashed green lines) where hydraulic measurements were conducted.

The laboratory experiments were conducted in a full-scale experimental model of a pool-type fishway (Fig. 1). The model structure was comprised of a steel frame with side panels of acrylic glass, allowing visualization of the fish movements occurring within the fishway. The flume was composed of six pools (1.9 m long×1.0 m wide×1.2 m high) divided by compact polypropylene cross-walls equipped with bottom orifices and surface notches. These were placed in a double offset arrangement, *i.e.*, each cross-wall had a bottom orifice on one side and a surface notch on the other, and this pattern alternated between pools. The flume tilted portion (10 m long) was set on an 8.5% slope, which is within the range of those commonly used for this type of fishway [25]. The flow entering the fishway was measured and regulated by a flow meter in the supply pipe. Finer adjustments were made by a valve located at the upstream end of the fishway, where a tank (1.5 m long×1.0 m wide×1.2 m high), separated from the flume by a mesh screen, allowed water to flow smoothly into the flume. The water level on the other hand was regulated by a slot gate located in the downstream end of the flume at a tank (4 m long×3 m wide×4 m high) where the flume starts. This tank is separated from the flume by a mesh panel and mimics the hydraulic environment found downstream of the entrance to the fishway allowing for better fish acclimation. By controlling the flow discharge and the level of water in each pool, either plunging or streaming flow could be produced (Table 1).

**Table 1:** Description of the two tested flow regimes.

Flow Regime	Q (L.s^−1^)	Orifice	Notch (width)	*h*m
Plunging	59.3	0.2 m×0.2 m	0.2 m	0.9 m
Streaming	78.5	0.2 m×0.2 m	0.2 m	1.0 m

Q – fishway discharge; *h*m – pool mean depth.

#### Fishway hydraulic measurements

Hydraulic conditions present at each pool have direct influence on the movements of fish [22], especially flow velocity and different turbulence parameters [19], [26], [27]. Therefore, in order to adequately characterize the hydraulics of the fishway, the three components of flow velocity (X, Y and Z) were measured with a Vectrino 3D ADV (Nortek AS) oriented downwards following a point-wise methodology. For this, a sampling point grid was created to cover the entire pool area with tighter spacing near the openings (5 cm), as these are areas of more pronounced velocity fluctuations, and wider spacing in the middle of the grid (10–15 cm). Velocity measurements were performed at a) two horizontal planes parallel to the flume bed (plane 1 at a height of 10 cm from the bottom, *i.e.* half the height of the bottom orifice and plane 2 at a height of *ca.* 78% of pool mean depth (*h*m), the plane directly affected by the surface notch), and b) two vertical planes, at the mid-width of each opening, parallel to the sidewalls (Fig. 1, Table 2). Measurements were performed at a 25 Hz rate for 90 s as this frequency and period were previously defined to be representative [28], at the second upstream pool. The hydraulic balance attained in the fishway allowed for any pool to be representative of every pool in the fishway. Instantaneous measures of velocity were filtered afterwards using the Goring and Nikora [29] phase-space threshold despiking method, modified by Wahl [30], using the WINADV freeware program [31]. Turbulence was characterized through Reynolds shear stress (RSS), which was calculated for the horizontal plane XY and for the vertical plane XZ, using the following formulas respectively:
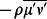
(1)

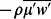
(2)where ρ = fluid density, *μ′* = fluctuating component of the velocity in the x direction, *v′* = fluctuating component of the velocity in the y direction and *w′* = fluctuating component of the velocity in the z direction.

**Table 2:** Point-grid information for the measurements performed with the Acoustic Doppler Velocimeter (ADV) at the 4 planes for each flow regime.

Flow Regime	Orientation	Plane	# points in the grid	Measured at
Plunging	Horizontal	1	97	10 cm from the bottom
Plunging	Horizontal	2	97	70.5 cm from the bottom
Plunging	Vertical	1	104	10 cm from the lateral wall adjacent to the notch inlet
Plunging	Vertical	2	104	10 cm from the lateral wall adjacent to the orifice inlet
Streaming	Horizontal	1	97	10 cm from the bottom
Streaming	Horizontal	2	97	78.2 cm from the bottom
Streaming	Vertical	1	105	10 cm from the lateral wall adjacent to the notch inlet
Streaming	Vertical	2	106	10 cm from the lateral wall adjacent to the orifice inlet

### Species Selection

The vast majority of studies on negotiating fishways use salmonids as target species. Advancing the scientific knowledge on navigating fishways requires the study of different non-salmonid species. Cyprinids are the dominant group of autochthonous freshwater fish in Mediterranean rivers ranging from large-size benthic potamodromous species to small-size pelagic ones, thus presenting different body shapes and occupying a range of ecological niches [32]. As studying the movements of all species would be time consuming, budget prohibitive and ultimately unfeasible, two species were chosen as representative of two morpho-ecological guilds [33]: the Iberian barbel (*L. bocagei*as the representative of large-bodied potamodromous benthic cyprinids, and the Southern Iberian chub (*S.pyrenaicus*), as the representative of small-bodied water-column resident cyprinids. The use of guilds, which represent groups of organisms independent of taxonomic envelopes that use the same range of resources [34], has been proposed as a tool for multi-specific approaches [35].

### Experiments

Two different flow regimes - plunging and streaming - were tested (Table 1). Three replicates of individual schools of 5 fish for each species were studied independently for each flow regime. Barbel and chub were captured on small coastal rivers from the Tagus river basin, central Portugal, during the migration season (Spring), by means of electrofishing using the procedures adopted by the European Committee for Standardization [23]. Fish were collected using a dip net and promptly placed in a mesh container fixed to the river bed at a location away from the influence of the electric field, allowing fish to be maintained in natural temperature and oxygenation conditions for the duration of the capture period. Fish were chosen to be of comparable length, and were transported to the laboratory in aerated containers filled with river water. Care was taken to reduce stress and expedite the procedure. At the laboratory, fish were stabilized in acclimation tanks (700 L) for at least 48 h before they were tested. Tests were performed in Spring during the migration season The quality of the water was examined on a daily basis and changed at a ratio of 150 L per day. Feeding stopped 24 h before each experiment. Fish were acclimatized to the fishway environment as for each experiment they were introduced in the tailwater-pool where the fishway openings were obstructed by a mesh panel that was removed after a period of 30 minutes. Each experiment lasted 90 minutes and was performed during the period of dusk and early night (17 h–01 h) to reflect the natural period of migration [36], [37]. Each fish was only used once and was allowed to ascend the fishway on its own volition. Two independent observers monitored the fish movements within the fishway. It was considered a “movement” whenever a fish negotiated a cross-wall in the upstream direction (a pool-to-pool displacement by one fish); it was considered a “success” when a fish reached the top of the fishway by negotiating the fifth cross-wall. All upstream pool-to-pool displacements performed by any fish in the school during the full length of the experiment (90 min) were registered. After each experiment fish were observed carefully to see if any injury, tissue damage, bruising and direct or delayed mortality was induced by excessive turbulence [38], [39], [40].

### Data Analysis

Differences in the number of upstream movements performed by the species during each flow regime - plunging and streaming - and through both opening types - bottom orifice and surface notch - were tested using proportion tests based on Chi-square distribution. The same procedure was applied to test for differences in the number of successful fishway negotiations attained individually and by both species in each of the flow regimes. To test the influence of flow regime, opening type and their interaction (flow regime×opening type) on the upstream movements of both species, a PERMANOVA test (permutation of residuals under a reduced model [41]) was applied. This statistical analysis is a powerful non-parametric approach that uses a permutational technique to enable significance tests for small sample sizes to be conducted [42] and was used to test the null hypotheses: (1) flow regime had no effect on the upstream movements of fish; (2) opening type had no effect on the upstream movements of fish; (3) effects of flow regime and opening types did not interact. Additionally, Mann-Whitney U tests and Sign tests were applied to the hydraulic data, to test for differences in flow velocities and turbulences (i.e. the Reynolds shear stress) between the two flow regimes. Chi-square proportion tests were performed in MedCalc software (MedCalc Software bvba). Mann-Whitney U tests and Sign tests were carried out in the software STATISTICA [43]. PERMANOVA tests were performed with the package PERMANOVA for PRIMER+v6.0 [44], [45].

## Results

### Hydraulics

**Figure 2:**
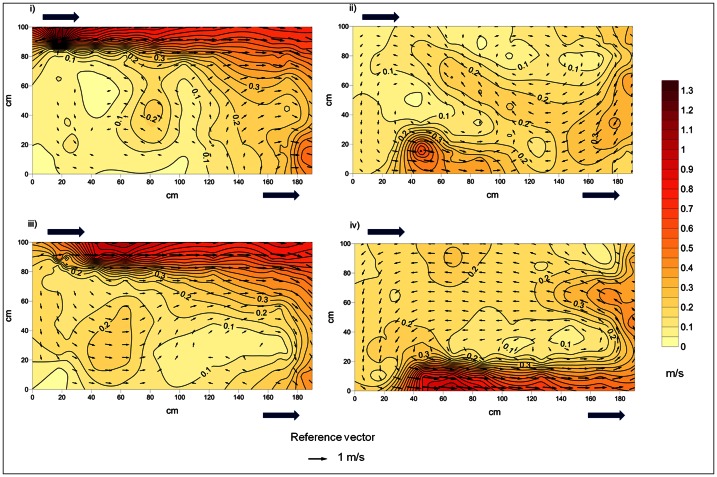
Velocity contour lines and vectors for the two horizontal planes in the two flow regimes. Plane 1 measured at 10 cm, Plane 2 measured at 78% pool mean depth (*h*m). Arrows within the diagram show the direction and magnitude of flow. Arrows outside of the diagram show the water inlet and outlet points. i) Plunging flow regime horizontal plane 1; ii) Plunging flow regime horizontal plane 2; iii) Streaming flow regime horizontal plane 1; iv) Streaming flow regime horizontal plane 2.

Figure 2 shows the flow velocity contours and vectors of both tested flow regimes at the two horizontal planes measured. On horizontal plane 1 of the plunging flow configuration, a jet-flow region could be distinguished from the water inlet to the opposite cross-wall. Horizontal plane 2 of this flow regime was characterized by a velocity peak close to the inlet of the water plunge that shaped a short downstream jet-flow region, interrupted by the main upstream flow. At horizontal plane 1 of the streaming flow regime, a large jet-flow region going from the water inlet to the opposite cross-wall could be observed. Horizontal plane 2 of this flow regime was characterized by a jet-flow from the water inlet to the opposite cross-wall. When compared, through a Sign test, the flow velocity pattern (XYZ resultant) in the two tested flow configurations differed on horizontal plane 2 (Sign test Z = 4.67, Non-ties = 97, p<0.001), closest to the water surface. Differences between flow regimes also arose in both horizontal planes, when the patterns of the horizontal and vertical components of Reynolds shear stress, were compared through Sign tests (Plane 1: RSS_xy_−Z = 3.66, Non-ties = 97, p-value <0.001; RSS_xz_−Z = 3.66, Non-ties = 97, p-value <0.001; Plane 2: RSS_xy_−Z = 2.64, Non-ties = 97, p-value = 0.008; RSS_xz_−Z = 3.45, Non-ties = 97, p<0.001).

**Figure 3:**
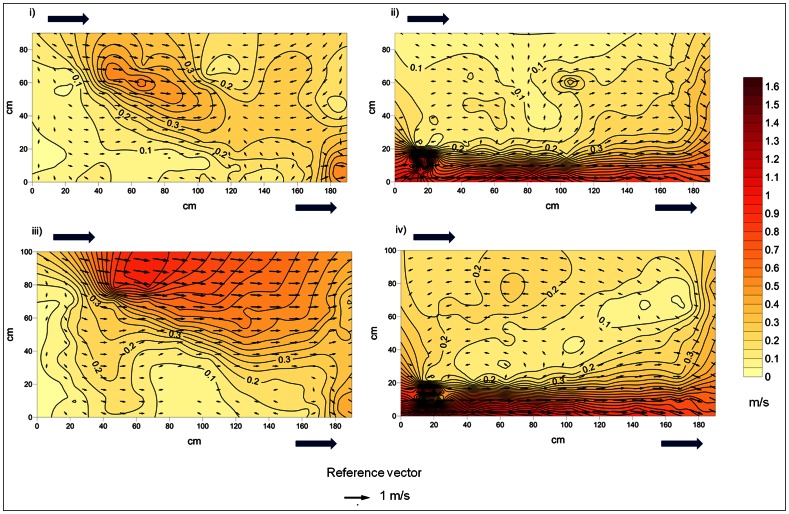
Velocity contour lines and vectors for the two vertical planes in the two flow regimes. Planes were measured at 50% of the orifice width. Arrows within the diagram show the direction and magnitude of flow. Arrows outside of the diagram show the water inlet and outlet points. i) Plunging flow regime vertical plane 1; ii) Plunging flow regime vertical plane 2; iii) Streaming flow regime vertical plane 1; iv) Streaming flow regime vertical plane 2.

Figure 3 shows the water velocity contours and vectors of both tested flow regimes at the two vertical planes considered. In the plunging flow, vertical plane 1, there was a noticeably high velocity plunge of water that was interrupted by an upstream flow. Vertical plane 2 of the same regime showed a strong jet stream region t from the water inlet to the opposite cross-wall. The streaming flow regime is characterized by a vertical plane 1 where the water flows, through the entire water column, from the water notch inlet towards the water outlet. Vertical plane 2 of this regime shows a strong jet stream from the water inlet up to the opposite cross-wall. When compared, through a Mann-Whitney U test, the water velocities (XYZ resultant) in the two tested flow regimes differed on vertical plane 1 (U = 4526, N_1_ = 104, N_2_ = 105, p = 0.033). This is the vertical plane which is under the direct influence of the surface notch inlet. In this vertical plane differences also arose when the same comparison was made for the Reynold’s Shear Stress horizontal and vertical components (RSS_xy_−U = 4343, N_1_ = 104, N_2_ = 105, p-value = 0.011; RSS_xz_−U = 4139, N_1_ = 104, N_2_ = 105, p = 0.0025).

### Fish

The fishway experiments showed that fish used the two opening types differently depending on flow regime and were closely related to the differences found in the hydraulic characterization (Table 3). During the plunging flow regime, 94% of all the upstream movements were conveyed through the bottom orifice opening (χ^2^ = 163.189, p<0.0001). A different result was observed for the streaming flow where the upstream movements were conveyed preferentially through the surface notch (57% ) while 43% occurred through the bottom orifice opening (χ^2^ = 10.208, p = 0.0014). The overall number of upstream movements between flow regimes was also different, with 74% of the movements occurring during the streaming flow regime (χ^2^ = 180.599, p<0.0001). This difference in the number of movements was also reflected in a higher number of successful fishway negotiations, with 74% (χ^2^ = 61.725, p<0.0001) occurring in the streaming flow regime.

**Table 3:** Number of upstream movements (i.e. upstream pool-to-pool displacements of a single individual) performed by all the individuals of each species through the bottom orifice and surface notch in the experimental pool-type fishway in both flow regimes (Plunging and Streaming).

Species	Flow regime	Bottom orifice	Surface notch	Total	# ofSuccesses	Totallength	Standard length	Body mass
Barbel	Plunging	37	1	38	15	26±3.9	20.6±3.4	181.2±88.0
	Streaming	71	83	154	48	25.5±2.3	22.7±2.9	239.6±26.0
Chub	Plunging	63	5	68	10	12.9±2.4	10.7±2.0	28.6±16.0
	Streaming	46	98	144	36	12.7±1.5	10.6±1.3	25.5±8.7

The number of successes (i.e. the number of times a fish attained the top of the fishway by the negotiation of the fifth cross-wall) achieved by all the individuals of each species in both flow regimes is also shown. The values of Total length (cm), Standard length (cm) and Body mass (g) are also presented (average ± standard deviation).

**Figure 4:**
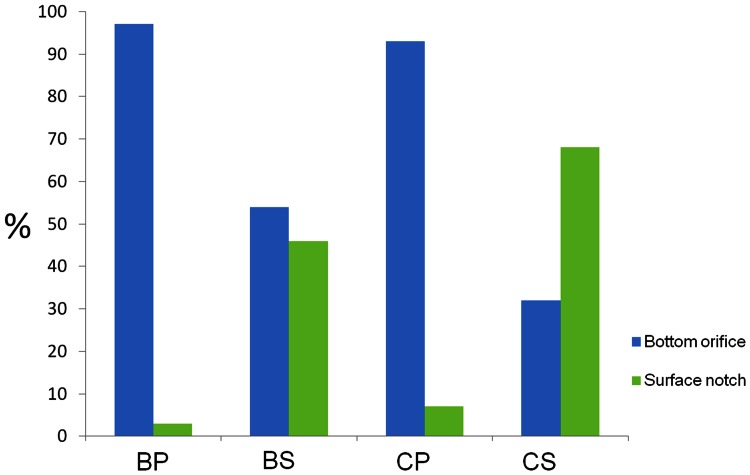
Proportions of upstream movements through both opening types by each species for both flow regimes. BP – Barbel in plunging flow regime; BS – Barbel in streaming flow regime; CP – Chub in plunging flow regime; CS – Chub in streaming flow regime.

The fishway experiments showed that the barbel used the facility differently for each distinct flow regime. Results from the PERMANOVA analysis (Table 4) showed a significant flow regime effect on the number of upstream fish movements within the fishway: overall, experiments conducted during streaming flow conditions revealed a higher proportion of barbel movements (80%) relative to plunging flows (20%). The effect of opening type alone was not significant, as the overall proportion of fish using notches (38%) and orifices (62%) were found to be similar. This produced a non-significant regime-by-opening type interaction. Experiments conducted during plunging flow conditions revealed an unequal proportion of movements through the orifices (97%) and notches (3%) (Fig. 4) (χ^2^ = 64.474, p<0.0001). During the streaming flow conditions, barbel tended to use the two opening types an almost equal percentage of times (χ^2^ = 1.571, p = 0.21), as 46% of the upstream movements were undertaken through the surface notch and 54% through the bottom orifice (Fig. 4). In fact, 99% of all barbel’s upstream movements through the surface notch were fulfilled in the streaming flow regime (χ^2^ = 132.250, p<0.0001). When analyzing the number of successes registered in each of the flow regimes there was evidence of higher success in the streaming flow regime (χ^2^ = 32.508, p<0.0001), which had over three times more successes (76%) than the plunging flow.

**Table 4:** Levels of significance of the two-factor PERMANOVA – permutation of residuals under a reduced model – testing the effects of flow regime plunging/streaming (Regime) and surface notch/bottom orifice (opening type) on the upstream movements of barbel and chub.

		Sum of Squares	Degrees of freedom	MeanSquares	Pseudo-F	p	Unique permutations
Barbel	Regime	1121.3	1	1121.3	11.317	0.01*	891
	Opening type	192	1	192	1.9378	0.272	917
	Regime×Opening type	48	1	48	0.48444	0.536	936
	Residuals	792.67	8	99.083			
Chub	Regime	481.33	1	481.33	11.46	0.008*	910
	Opening type	3	1	3	0.00714	0.792	927
	Regime×Opening type	1008.3	1	1008.3	24.008	0.006*	921
	Residuals	336	8	42			

* Significant with an α = 0.05.

The fishway experiments showed that the chub used the facility differently for each distinct flow regime. Results from the PERMANOVA analysis showed that flow regime had a significant effect on the number of fish upstream movements within the flume: in general, experiments performed in streaming flow regime revealed a higher ratio of chub movements (68%) relative to plunging flow regime conditions (32%). The isolated effect of opening type was not significant, because the overall proportions of fish using the notch opening (49%) and the bottom orifice (51%) were found to be equivalent. Nevertheless, a significant interaction between regime and opening was detected (p<0.01), indicating thereby that the flow regime affected the number of upstream movements differently, depending on the type of cross-wall opening. Accordingly, tests conducted in plunging flow regime revealed an uneven proportion of movements occurring through the bottom orifice (93%) and surface notch (7%) (Fig. 4) (χ^2^ = 95.559, p<0.0001). As a result, the bulk (58%) of the upstream movements undertaken by chub through the bottom orifice were completed during the plunging flow condition (χ^2^ = 4.697, p = 0.0302). In contrast, chub were more likely to use the surface notches (68%) for streaming flows to the detriment of bottom orifices (32%) (χ^2^ = 36.126, P<0.0001), and, as a result, 95% of all upstream movements through the surface notch were accomplished during streaming flow conditions (χ^2^ = 164.349, p<0.0001). When analyzing the successes registered in each of the flow regimes there was evidence of higher success achieved during streaming flow conditions (78%) relative to plunging flows (χ^2^ = 27.174, p<0.0001). After the experiments, fish did not present any signs of injury produced by turbulence.

## Discussion

The morphology of a fish species, its body size and shape [15], [46], [47] can determine the swimming ability and hydraulic suitability of the species to a specific environment [33]. This study allowed the assessment of movements and navigation behaviour of cyprinid species representing different morpho-ecological guilds (benthic/potamodromous *vs*. water-column/resident) within a full-scale model of a pool-type fishway with different flow regimes. It contrasts with the majority of studies on fish transfer devices that often lack balanced experimental designs. It overcomes study limitation conducted in the wild by enabling the variables of interest to be manipulated while controlling for confounding effects [21]. The importance of this study is highlighted by the fact that it is the first study that examines and compares the movements and navigation behaviour of two morpho-ecologically different fish species in a pool-type fishway with streaming or plunging flow. The vast majority of studies have neglected the streaming flow regime of pool-type fishways and only studied the plunging flow regime [18]. The philosophy behind fishway research, design, and construction has evolved over the years and has been moving towards more holistic fishways, i.e. fishways that can serve a wide spectrum of species with different ecological niches. By focusing on the nature of movements of different species guilds within a fishway, this work moves a step forward towards holistic multi-species fishways.

The upstream movements of any fish are, as those of other animals [48], [49], always non-random and are a consequence of colonization, reproductive or feeding needs. During the experiments both fish species showed to be able to negotiate the fishway in both flow regimes. After the experiments, fish did not present any signs of injury produced by turbulence, indicating that fish probably limited their exposure to high RSS and flow velocities to a minimum.

Recent studies have defined the critical swimming speed of barbel [50] –0.81 m.s^−1^ (3.1 BL. s^−1^) – and of chub’s sister species the bordallo (*Squalius carolitertii* (Doadrio, 1988)) [51] –0.54 m.s^−1^(4.4 BL. s^−1^). The flow velocity in the bulk of the pool area for the two flow regimes never surpassed 0.3 m.s^−1^ attaining only higher velocities near the water inlets. This demonstrates that the velocity fields in both flow regimes were acceptable for these species and allowed fish to recover from negotiating the cross-wall where they had to undertake burst movements to offset the high flow velocities found at the cross-wall openings.

The findings of this study showed that during the plunging flow regime, the surface notch was seldom used by either species, which was not surprising since cyprinids are known to have a limited leaping ability [52]. An opposite trend was found for a salmonid species, the Atlantic salmon (*Salmo salar*), that preferred to use the surface notch opening for its upstream movements in an experimental pool-type fishway during plunging flow regime [53]. Guiny et al. [54] studied salmonids (Atlantic salmon parr and brown trout (*Salmo trutta*)) and found that both species used almost exclusively the bottom orifice to negotiate a cross-wall. It is important to note though that in the Guiny et al. [54] experiments the water column below the cross-wall was just 20 cm deep, and with this reduced depth the plunging water touched the flume bed and rebounded. This effect created flow vectors moving in different directions and may have reduced fish movements through the surface notch. In the present study, the vast majority of upstream movements during plunging flow by both tested species occurred through the bottom orifice. This result is in line with the findings of Silva et al. [55], where barbel also preferred to use the bottom orifices in contrast to the surface notch opening type for their upstream movements during plunging flows. Ficke et al. [56] also concluded that pool-type fishways that require fish to leap over the crest of the surface notch and instream structures that produce a vertical drop of more than 10 cm will not allow significant upstream passage of small-sized fish species. In the current study, only a few individuals of both species, most likely those with stronger swimming capacity, were able to negotiate the pool through the surface notch during plunging flow. These results confirm the hypothesis that species would not be able to use the surface notch during plunging flow, and will be forced to use the bottom opening. On the other hand, streaming flow facilitated access to the surface notch, allowing species to move upstream more successfully with fewer constraints and possibly with less energy expenditure, increasing the number of movements. By turning the surface notch into a more accessible opening type, the streaming flow regime increased the area available for negotiating cross-walls. However, species displayed different choices of opening types for their upstream movements. These preferences are congruent with their different morpho-ecologic guilds: the chub, a water column species, had a clear preference for the surface notch opening. In contrast, the barbel, a bottom oriented species, divided their upstream movements between opening types, demonstrating that this is a plastic species with the ability to use the entire water column.

The positive rheotactic behaviour during their upstream migrations [22], [52] guides fish upstream by orienting them against the downstream flow. Fish may also display rheotactic responses to turbulence promoting migratory movements [22], [57]. Based on these facts, the hydraulics of the two tested flow regimes allowed a simple explanation of the experimental results. The plunging flow regime provided little or no attraction to the surface notch, since an upstream flow circulation was generated which interrupted the downstream flow possibly miss-orientating fish. The same does not happen for the streaming flow, where there was a continuity of downstream flow allowing fish to be attracted to the surface notch. Looking at vertical plane 1, this pattern becomes even more evident. In streaming flow there was continuity of flow in the entire length of the pool and throughout the pool water column. This is in contrasting with the sudden change in flow direction observed in vertical plane 1 of the plunging flow regime. The fact that the flow regimes differed in their RSS patterns also supports the difference in movements and successes attained in both flow regimes. This proves that not only velocity but also turbulence parameters can explain fish preferences when navigating through a fishway [19], [57].

Streaming flow proved to be a more efficient flow regime by enhancing upstream movements, facilitating the movement of the surface oriented species and increasing the options of the bottom oriented species. Through a more suitable hydraulic environment, streaming flow increased the negotiation success of both species. Thus, the streaming flow configuration is the most suitable for fishways constructed in river systems in which a wide range of fish morpho-ecological traits are found. Existing fishways should be retrofitted to accommodate the behaviour and swimming capabilities of the species present at the site [58]. The present study shows that enabling streaming flows on future and on existing fishways is desirable. This must be considered during fishway design to allow this flow regime to be attained even with low flows. Future studies should concentrate on the behaviour of other non-salmonid species in pool-type fishways. Focusing on the swimming capabilities of different species, finding the best fishway configuration that maximizes attraction [18], [59], [60], keeping velocity gradients [22] and turbulence levels acceptable [19], ensuring negotiation success of all fish species present in the target system, and minimizing fishway selection of species, size-classes and ages [61]–[64] is the best way to move towards the objective of an holistic fishway that accommodates the entire range of species present at a given system.
